# Supplementary material for: An exploratory study of the impact of CT slice thickness and inter-rater variability on anatomical accuracy of malunited distal radius models and surgical guides for corrective osteotomy
Source: PLoS One. 2024 Oct 10;19(10):e0311805. doi: 10.1371/journal.pone.0311805 (PMC11476685; doi:10.1371/journal.pone.0311805)
Supplement: S1 File — (DOCX) [file pone.0311805.s001.docx]

S1 Table 1. Q-Q plots for differences between the average inter-rater guide surface discrepancy and surface discrepancy from the reference radius for guides created for each CT slice thickness and each participant.

| 1mm: | 1.25mm: |
| --- | --- |
| 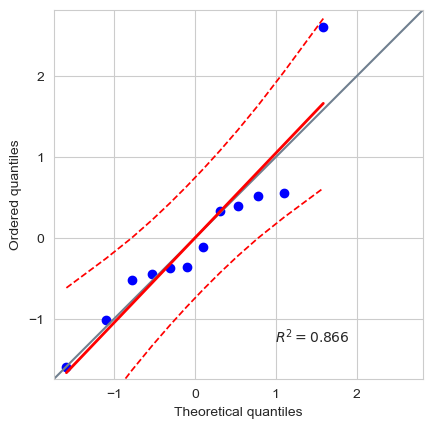 | 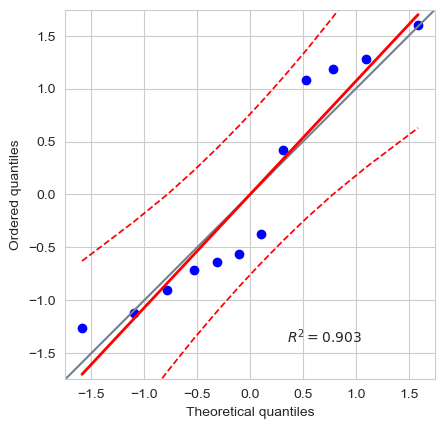 |
| 1.5mm: | 1.875mm: |
| 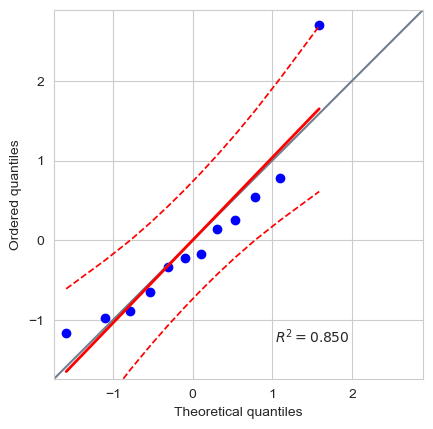 | 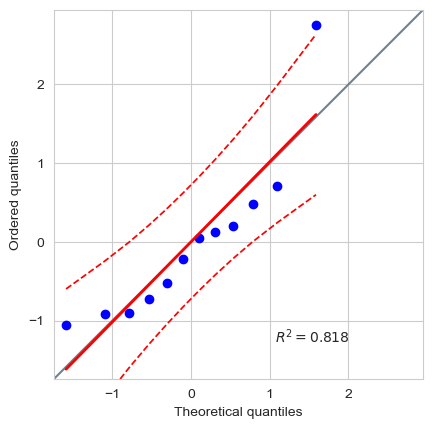 |
| 2.5mm: |  |
| 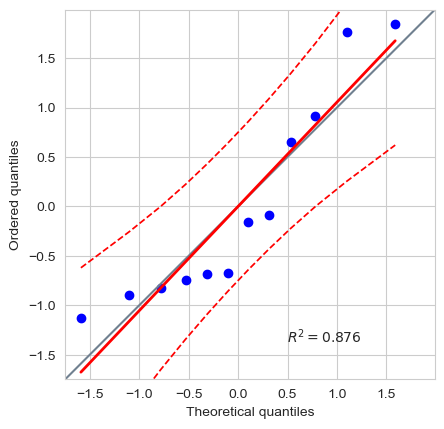 |  |

Table 2. Q-Q plots for differences between the average inter-rater radius surface discrepancy and surface discrepancy from the reference radius for radiuses created for each CT slice thickness and each participant.

| 1mm: | 1.25mm: |
| --- | --- |
| 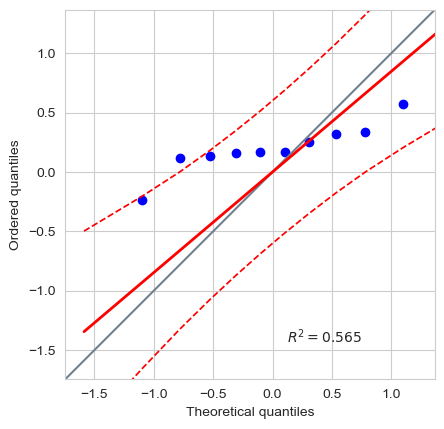 | 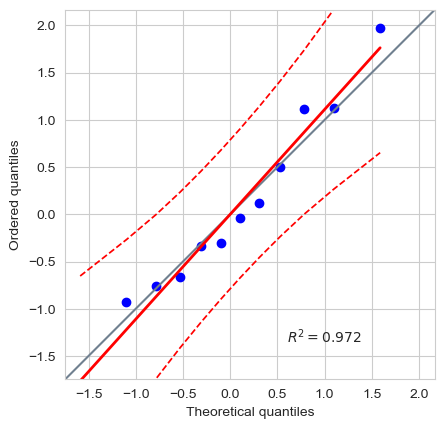 |
| 1.5mm: | 1.875mm: |
| 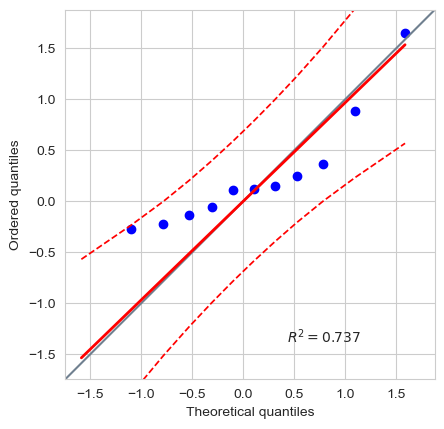 | 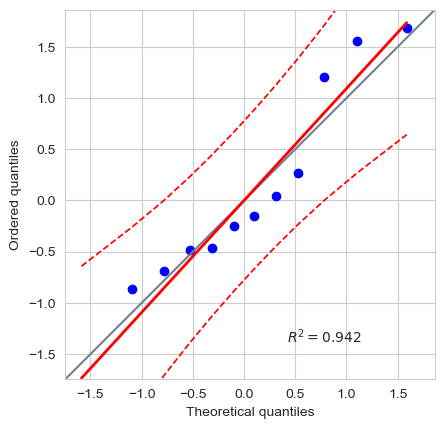 |
| 2.5mm: |  |
| 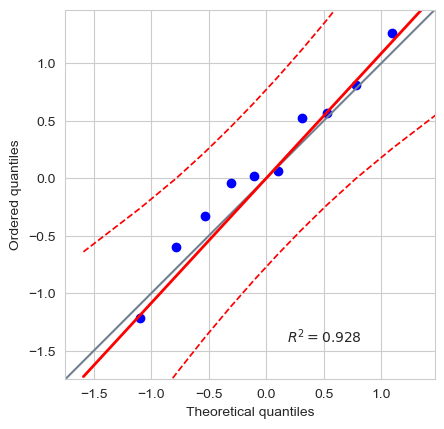 |  |
