# Supplementary material for: An exploratory study of the impact of CT slice thickness and inter-rater variability on anatomical accuracy of malunited distal radius models and surgical guides for corrective osteotomy
Source: PLoS One. 2024 Oct 10;19(10):e0311805. doi: 10.1371/journal.pone.0311805 (PMC11476685; doi:10.1371/journal.pone.0311805)
Supplement: Attachment — Submitted filename: Response to reviewers.docx [file pone.0311805.s003.docx]

Dear Editor and Reviewers,

We want to thank the reviewers for taking the time to review our manuscript; we appreciate the insightful and constructive comments. We believe that the manuscript was improved after we supplied the manuscript with the reviewer’s suggestions. In the following, we address all comments made by the editor and the reviewers. Changes to the manuscript are tracked, and comments are added in the manuscript to indicate which reviewer’s comment a given change refers to.

Editor’s comments:

1. Please ensure that your manuscript meets PLOS ONE's style requirements, including those for file naming.

Authors’ response: We revised our submission and the manuscript to ensure that it meets PLOS ONE’s requirements.

2. Thank you for stating the following financial disclosure:

“The study was financed by grants from the Swedish state under the agreement between the Swedish government and the county councils, the ALF-agreement (ALFGBG-966260).”

Please state what role the funders took in the study.  If the funders had no role, please state: "The funders had no role in study design, data collection and analysis, decision to publish, or preparation of the manuscript."

Authors’ response: The funders had no role in the study, and the above statement should be added to the financial disclosure.

3. We are unable to open your Supporting Information file [data_raw.zip]. Please kindly revise as necessary and re-upload.

Authors’ response: We apologise for that. The original data are quite large and were provided in a .pkl format that is easy to open as a data frame using *pandas* library in Python. We now supplied the supporting information with the raw data available in text format so that it can be opened in other programs.

4. Please review your reference list to ensure that it is complete and correct. If you have cited papers that have been retracted, please include the rationale for doing so in the manuscript text, or remove these references and replace them with relevant current references. Any changes to the reference list should be mentioned in the rebuttal letter that accompanies your revised manuscript. If you need to cite a retracted article, indicate the article’s retracted status in the References list and also include a citation and full reference for the retraction notice.

Authors’ response: We reviewed the reference list and formatted the citing style, so it meets PLOS ONE’s requirements

5. Review Comments to the Author

Reviewer #1: The paper An exploratory study of the impact of CT slice thickness and inter-rater variability on anatomical accuracy of malunited distal radius models and surgical guides for corrective osteotomy is well written and documented. The aim was three-folded. Images are of high quality. Results and conclusions are clear. References are up to date.

Authors’ response: We thank the reviewer for the positive feedback on our manuscript.

Reviewer #2: The paper is very well-written, and the studies are well-designed. I have the following comments:

1. The Hausdorff distance and the ADE are absolute measures. It is true that absolute error is important in determining guide fit, however, it is possible that physically larger guides in larger patients will contribute more to the overall error in a study. That is, the composition of the patient cohort might affect the inference of the study. Hence, it might be useful to also report relative errors, i.e., absolute errors normalized by total volume, so that results are comparable across patient cohorts in future.

Authors’ response: Although this is an interesting thought, in case of the guide fit, we doubt that the size of the bone will influence the fit of the guide as measured by Hausdorff distance. We chose this metric as one localised overlap between the guide and the bone will have the same consequence for the guide fit, regardless of the size of the bone. Normalising the Hausdorff distance with bone volume could, however, incorrectly indicate a more serious negative effect on the guide fit in a smaller bone.

Regarding the ADE, there could be a difference between larger and smaller bones, since relatively more detail is lost if the details are relatively smaller. However, as we stated in the discussion, we suspect that this metric may not be sensitive enough metric to establish the largest CT slice thickness suitable for 3D VSP. We agree, though, that the impact of bone size, e.g. in children or non-Caucasian populations should be accounted for in future studies. We mention the homogeneous bone size in our cohort as a limitation of this study:

“The size variation of the assessed bones was also low in the cohort and therefore we could not assess whether the results depend on the bone size.”

And in the last paragraph in response to Comment #5:

“We also suggest using a different metric than ADE to assess the suitability to the models for 3D VSP and normalise the results with respect to the bone volume, so the results are comparable across various cohorts.”

2. It would be useful to have a comment on the effects of image acquisition parameters that might affect the value of the optimal CT slice thickness discovered in this study.

Authors’ response: Thank you for this suggestion. We added a relevant paragraph in the Discussion:

“Corroborated by larger-scale studies, our results could be used to adjust CT acquisition and reconstruction parameters for 3D VSP and PSSG design. During acquisition, the parameter that can be adjusted to decrease the slice thickness is the helical pitch. Increasing the pitch will result in a lower resolution but also decreased radiation exposure and time of the examination. Once the data is acquired, the final image resolution can be adjusted during reconstruction. Decreasing image resolution during reconstruction will result in less noisy image. Lower-resolution images also take up less space. Even though this is rarely considered when optimising imaging protocols, virtual storage has a cost. Our and future results should guide medical physicists in designing new protocols to optimise resource use and patient safety.

3. Do the authors expect that the optimal CT slice thickness would be affected by demographic parameters, e.g., age, sex, given the anatomical differences associated with the same?

Authors’ response: We thank the reviewer for again bringing up a relevant point. We know from experience that image segmentation of arthritic and osteoporotic bone is difficult, and we suspect that the difficulty would increase with increased CT slice thickness. We know also that arthritis incidence increases with age and in older cohorts, women are more at risk. Given that our cohort mostly consisted of older women, we don’t have enough data to investigate the impact of the demographic parameters on the results. We added this to the limitations section in the manuscript:

“Furthermore, our cohort included only one man, and the median age of the participants was above 60 years old. The size variation of the assessed bones was also low in the cohort and therefore we could not assess whether the results depend on the bone size. Based on the limited, demographically homogeneous cohort, we also could not assess the impact of age or sex on the results. “

4. In the radius-radius comparison, it would be interesting to split the error into guide-contact region (distal radius and partial shaft) vs non-contact region (proximal radius and shaft). It is possible that given the high variations in small-scale structures in the radial head and neck, the clinically acceptable value discovered for the guide-contact region might be even lower than presented.

Authors’ response: We agree that this information would make our findings more interesting. We now added the results of radius models discrepancies split between the distal and the proximal parts in figure 4, as well as in the manuscript:

“An additional analysis of the radius model (radius-to-radius) was also conducted for the whole radius and individually for the distal and proximal parts. Each radius model was split by a plane perpendicular to the long axis of the bone positioned directly below the proximal edge of the guide base. “

“For the distal radius, it (ADE) was 0.025mm [95% CI: 0.023 -0.027] and for the proximal part: 0.03mm [95% CI: 0.22 -0.037].Since we could not assume a normal distribution for the Bland-Altman analysis in two out of five groups (S2 Table), we show the distribution of the absolute ADE for every pair of higher-resolution models compared to every rater’s reference model, separately for the distal and proximal parts (Fig 4). The median absolute ADE is generally larger than the average inter-rater variability for all CT slice thicknesses and both the distal and proximal parts. Overall, we observed larger ADE values for the distal part than the proximal part. The median absolute ADE created from CT with a slice thickness of 1.25m and 1.5mm are comparable. Much larger absolute ADE and data spread are observed for models created from CT images with a slice thickness of 1.875mm and 2.5mm, especially for the distal radius.”

**Fig 4. Distribution of absolute ADE for radius models separated into the distal (dist) and proximal (prox) parts, created from CT images with a slice thickness of 1mm, 1.25mm, 1.5mm, 1.875mm, and 2.5mm compared to each rater's (EG, KL, CS) reference. The dashed lines indicate the average inter-rater absolute ADE. One outlier of ADE > 0.65 for CSv2.5mm not shown.**

5. Please add a comment on factors that might contribute to different results in a large-scale study as compared to the exploratory study.

Author’s response: We added the following paragraph in the Discussion:

“A larger study is necessary to perform equivalence tests of models for 3D VSP and PSSG design created from CT images with larger slice thickness to those currently used. Ideally, images and VSPs from multiple centres would be acquired to further study the impact of a CT machine on the images, and various raters on the segmentation. A more varied and larger cohort would further allow to investigate other factors that could influence the optimal imaging protocol, like age and age-related conditions, like arthritis, ethnicity, or sex, mentioned in the limitations section. We also suggest using a different metric than ADE to assess the suitability to the models for 3D VSP and normalise the results with respect to the bone volume, so the results are comparable across various cohorts. Intra-articular and diaphyseal malunions should also be considered. The results may also vary for other anatomies.”
